# The Alcohol and Epoxy Alcohol of Zingiberene, Produced in Trichomes of Wild Tomato, Are More Repellent to Spider Mites Than Zingiberene

**DOI:** 10.3389/fpls.2020.00035

**Published:** 2020-02-21

**Authors:** Mohammad H. Dawood, John C. Snyder

**Affiliations:** ^1^ Department of Horticulture, University of Kentucky Lexington, Lexington, KY, United States; ^2^ Department of Horticulture and Landscape, University of Kufa, Najaf, Iraq

**Keywords:** breeding, insect resistance, 7-epi-zingiberene, 9-hydroxy-zingiberene, tomato, mite repellency, antixenosis, trichome

## Abstract

Allelochemicals that are present in trichome secretions of wild tomato species play a major role in mediating interactions with arthropods, often conferring a high level of resistance *via* antibiosis and antixenosis. Many accessions of the wild tomato relative, *Solanum habrochaites* (*S.h*), possess high levels of resistance to arthropods. The monocyclic sesquiterpene hydrocarbon, 7-epi-zingiberene, is a major defensive component found in trichome secretions of certain accessions of *S.h*. We have used LA2329, an *S.h*. accession, as a donor in a breeding program designed to introgress zingiberene into cultivated tomato. However, the composition of trichome secretions in our population of LA2329 is segregating, with some individuals producing mainly 7-epi-zingiberene in their secretions while others producing two additional, unidentified compounds in their trichome secretions. To investigate if these other compounds may also contribute to arthropod resistance, trichome secretions were collected from plants of *S.h* LA2329 grown under greenhouse conditions and then major compounds were isolated by silica gel column chromatography and tested for their ability to repel two spotted-spider mite (TSSM), *Tetranychus urticae*. Isolation and identification of allelochemicals were aided by use of gas chromatography/mass spectroscopy. The results revealed the presence of three predominate chromatographic peaks: 7-epi-zingiberene, 9-hydroxy zingiberene, and 9-hydroxy,10,11-epoxy-zingiberene. Results of testing isolated compounds for repellency to TSSM using bridge bioassays revealed that the repellent activities of 9-hydroxy zingiberene and 9-hydroxy,10,11-epoxy-zingiberene were each significantly higher than that for 7-epi-zingiberene. These results support the idea that the degree of repellency may differ among plant allelochemicals and also emphasize the potential value of introgressing the presence of 9-hydroxy zingiberene and 9-hydroxy,10,11-epoxy-zingiberene into cultivated tomato to enhance its arthropod resistance.

## Introduction

The tomato, *Solanum lycopersicum,* is one of the most widely grown and important vegetables produced worldwide (Dorais et al., 2008). Numerous factors can affect tomato productivity dramatically. One of these factors is arthropod infestation, resulting in direct damage and/or disease transmission (Nault and Speese III, 2002). Most cultivated tomatoes are susceptible to diseases and pests, leading growers to apply pesticides and insecticides (Letourneau and Goldstein, 2001) to control these problems. Breeding for resistance may provide an alternative to extensive pesticide use.

Tomato has been greatly improved by introgression of disease resistance genes from wild relatives (Hajjar and Hodgkin, 2007; Firdaus et al., 2012). There are wild tomato accessions that are highly resistant to a wide range of arthropod pests (Freitas et al., 2002), but introgression of arthropod resistance into commercially accepted tomato varieties remains an unfulfilled goal.

High levels of resistance to arthropods exist in several wild species of tomato and in many cases, resistance has been associated with trichomes and trichome secretions (Snyder et al., 1993; Schilmiller et al., 2009; Ekanayaka et al., 2014). For example, acyl sugars secreted by trichomes on *S. pennellii, S. galapagense,* and *S. pimpinellifolium* have been associated with resistance to an array of arthropods (Shapiro et al., 1994; Liedl et al., 1995; Mutschler et al., 1996; Alba et al., 2009; de Resende et al., 2009; Dias et al., 2013). Methyl ketones, primarily 2-tridecanone have been linked to arthropod antibiosis displayed by certain accessions of *S. habrochaites (S.h)* (Alba et al., 2009). For other accessions of *S.h*, resistance has been attributed to the presence of sesquiterpenes in their trichome secretions (Carter et al., 1989a; Weston et al., 1989; Eigenbrode et al., 1994; Chatzivasileiadis and Sabelis, 1997).

It has been known for more than 25 years that trichomes on certain accessions of *S. h*. were associated with the production of sesquiterpenes and that there was structural diversity among sesquiterpenes produced (Guo et al., 1993). Additionally, oxygenated sesquiterpenoids, mainly carboxy acids of sesquiterpenes have been documented in *S.h*. often as predominate components of trichome secretions (Coates et al., 1988; Snyder et al., 1993). More recently diversity and details of terpene production, especially those terpenes present in trichome secretions has been elucidated in *S.h*. Much of the chemical diversity of trichome secretions in *S. h.* has been associated with sequence variation of sesquiterpene or monoterpene synthases associated with the TPS20 locus located on chromosome 8 of tomato. All of these enzymes utilize cisoid substrates, either neryl diphosphate for monoterpene production or 2z,6z-farnesyl diphosphate for sesquiterpene production (van der Hoeven et al., 2000; Besser et al., 2009; Sallaud et al., 2009; Schilmiller et al., 2009; Falara et al., 2011). 

There is an extensive collection of wild tomato germplasm curated by the Tomato Genetics Resource Center (TGRC), located in Davis, California. Over a period of many years, *S.h*. has been systematically collected over its natural geographic range. This extensive and valuable resource is maintained for researchers worldwide. Gonzales‐Vigil et al. (2012) have surveyed 79 accessions of *S.h*. from the collection at TGRC for diversity of trichome-associated terpene production. Trichome secretions on these accessions were predominated either by sesquiterpenes or by monoterpenes. 21 different terpenes were detected among these 79 accessions. And the accessions could be grouped by the predominate sesquiterpene present in the trichome secretions. For example, trichome secretions on more than ½ of the accessions were predominated by 7-epi-zingberene **[1]**. The sesquiterpene gamma-elemene was predominate on 19 accessions, beta caryophyllene was predominate on nine accessions and trichome secretions on 8 of the 79 accessions were predominated by monoterpenes, either β-phellandrene or limonene. How this chemical diversity may relate to arthropod resistance has not been well characterized

Among accessions, there is an array of sesquiterpene hydrocarbons produced by trichomes on *S.h.* (Ekanayaka et al., 2014). However, the presence of 7-epi-zingiberene **[1]** has frequently been associated with pest resistance (Carter et al., 1989b; Antonious and Kochhar, 2003; Bleeker et al., 2012; Álvarez Gil, 2015; de Oliveira et al., 2018). Recently at the University of Kentucky, we have been attempting to introgress the presence of high levels of **[1]** and high type IV trichome density from *S.h.* LA2329 into cultivated tomato. We have been successful in the introgression of **[1]** from wild to cultivated tomato, and currently possess several advanced breeding BC3F7 lines having yields and seed set similar to the recurrent parent with levels of **[1]** similar to that in the wild donor parent (Snyder et al., 2018). As part of this breeding program we have worked extensively with the *S.h.* donor line LA2329. Associated operations have included seed increase and monitoring production of **[1]**. Several years ago, we discovered that the composition of trichome secretions varied within our population of LA2329, with some plants having one major component and others having three major components as determined by gas chromatography. All plants produced **[1]**, but some of the plants also produced two additional abundant compounds of unknown identity that were later eluting than **[1]** when separated by gas chromatography. We have maintained these two distinct chemotypes by selfing or sib-mating within chemotypes. Leaves of the two chemotypes appeared to be equally resistant to spider mites when bioassayed. However, after an unintended infestation of whiteflies *(Bemisia* spp.) in our greenhouse we observed that the plants having the three major compounds were less preferred than those with **[1]** alone. This difference in whitefly preference between chemotypes could have been related to quantitative or qualitative chemical differences in the chemotypes, or to other, unmeasured differences.

Based on these observations, we were particularly interested in assessing the potential for qualitative differences of antixenotic activity of the three major compounds present in LA2329. Because the bridge bioassay has the ability to demonstrate differences in antixenotic activity or repellency to spider mites among closely related compounds (Snyder et al., 2011), the goal of this research was to isolate and evaluate the spider mite repellency of the three major compounds present in trichome secretions of LA2329. We wanted to know whether these three compounds differed in their degree of repellency to spider mites.

## Materials and Methods

### Biological Materials

Seeds of *S.h.* accessions LA2329 and LA2167 were obtained from the Tomato Genetics Resource Center, Davis, CA and seeds of PI127826 were obtained from USDA-ARS, Geneva NY. After seedling establishment, plants were propagated by cuttings or from seed produced by selfing or sib-mating. Plants were grown in the greenhouse at the University of Kentucky, using Pro Mix BX (Premier Tech Horticulture, Quakertown, PA) as growing medium. Plants were irrigated with a fertilizer solution containing Peter’s Professional 5-11-26 (ICL SF USA & Canada, Dublin, OH) plus CaNO_3_ (Viking Yara, Tampa, FL) to provide 180 ppm of N. Plants were grown during spring, summer, and fall, under natural daylength conditions. The average temperature and relative humidity of the greenhouse during this time period were 24°C, and 67% RH, respectively. Spider mites, *Tetranychus urticae* (TSSM) were reared and handled using procedures as outlined by Snyder et al. (2011).

### Leaflet Wash Preparation for Determination of Qualitative and Quantitative Variation of Trichome Secretion Components

We investigated quantitative and qualitative variation of abundance of each major component of trichome sections on plants of the two chemotypes of LA2329 (one plant of each chemotype) and on LA2167 and PI127826 (one plant of each accession). To prepare leaflet washes the center 1/3 portion of each of three leaflets from the third leaf position of a plant was placed into a 20 ml vial and then 2.0 ml of *n-*hexane was added. The vials were vortexed for 30 s and then the extract was analyzed by GC-FID using procedures outlined below. The area of extracted leaf tissue was determined by using the open-source image analysis software ImageJ/Fiji (ImageJ 1.47v, National Institutes of Health, USA). Results were expressed as FID detector response (area units) per cm² of leaflet tissue. For this experiment each plant was sampled three times.

### Oleoresin Preparation for Bioassay and Chromatography

To prepare oleoresins of the two chemotypes of LA2329 for bioassays, leaflets, usually from the third or fourth leaf from the apex, were removed from plants and were then steeped in *n*-hexane for 30 min using approximately 1 ml of *n*-hexane per leaflet. Leaflets were then removed, and hexane was removed by use of a N_2_ stream. Oleoresins were stored at 3°C and for bioassay, they were diluted in hexane.

To prepare extracts for subsequent open column chromatography, two Kg of leaflets from the LA2329 chemotype that contained three major components (chemotype A) were collected in a 10 L beaker. Leaflets were soaked in an excess of *n*-hexane. After stirring, leaflets were removed, and the extract was filtered (Whatman 934-AH). Subsequently over a period of several days the *n*-hexane was allowed to evaporate under a chemical hood. After that, additional *n*-hexane was eliminated from the oleoresin by use of a gentle stream of N_2_. This extract was weighed and stored in a refrigerator at 3°C.

### Gas Chromatography

A gas chromatograph, Hewlett Packard 5890 Series II, equipped with a flame ionization detector (GC-FID) and an RTX-5 column (5% diphenyl/95% dimethyl polysiloxane, 15 m, 0.53-mm ID, 0.5 µm, Restek Corporation, Bellefonte, PA) was operated as follows: injector 250°C, detector 300°C, oven initial temperature 50°C for 1 min, then increasing at 20°C/min to 260°C. Helium was the carrier gas flowing at 14 ml/min. Tetradecane (Acros Organic^®^, New Jersey, USA) served as the external standard to verify retention time and detector response. Calculation of the concentration of components in trichome secretions was based on a detector response or on a tetradecane standard curve.

Gas chromatography-mass spectroscopy (GC-MS) was used to verify identities of **[1]** 7-epi-zingiberene**, [2]** 9-hydroxy zingiberene, and **[3]** 9-hydroxy,10,11-epoxy-zingiberene in leaflet washes of LA2329. Identification was based on library spectra, published spectra (Anonymous, 2017) as well as on spectra obtained from leaflet washes of the *S.h* accession LA2167, the source material used by Anonymous (2017) to identify **[2]** and **[3]**. The GC-MS was an Agilent model 6890N equipped with an Agilent 5975 mass selective detector and 7673B injector. Oven temperature was programmed from 50°C to 250°C at a rate of 20°C/min (1 min initial hold). Injector temperature was set at 250°C and 300°C, respectively. A 30 m × 0.25 mm ID DB-5 capillary column (0.25 µm film thickness) was used. Helium was a carrier gas at 1.0 mL/min). AMDIS 32 [US National Institute for Standards and Technology, Gaithersburg, MD, USA] was used to extract and compare mass spectra.

### Silica Gel Chromatography

To separate components of the oleoresin, open column chromatography was used. A glass column (20 × 0.8 cm) was filled with SiO_2_ (230–400 mesh, Natland International Corporation, NC, USA) in the usual fashion. 100 mg of oleoresin was loaded onto the column which was then eluted with *n*-hexane: methyl-tert-butyl ether (MTBE). Ratio of *n*-hexane:MTBE ranged from 97:3 to 75:25. Two ml fractions were collected, and elution was monitored by spotting one drop of each fraction onto a thin layer chromatography plate (Silica gel 60 A with fluorescent indicator, Whatman Int Ltd, England) followed by illumination with 254 nm ultraviolet light (UVGL-25, Mineralight Lamp, UVP Inc, CA, USA). Composition, concentration and purity of each uv-positive fraction were determined by GC-FID. For factions chosen for bioassay, solvent was evaporated by a stream of N_2_, and the residue was dissolved in *n*-hexane.

### Bridge Bioassay

The bridge bioassay was deployed as described by Snyder et al. (2011) and Guo et al. (1993), which is a modest and easy technique for testing TSSM repellency of natural products. The bioassays were conducted on a lab bench. Temperatures averaged about 21C and varied ±2 degrees during days the bioassays were conducted. To provide clarity for our results, a brief overview of the principle aspects of the bioassay is appropriate. The bioassay arenas (**Figure S1**) consisted of two small rectangles (1 × 0.75 cm each) of ﬁlter paper held horizontally in a clamp. One rectangle was treated with a known concentration in *n*-hexane (20 µL/0.75 cm² of filter paper). The other filter paper rectangle was treated with *n*-hexane only as a control. After the *n*-hexane evaporated, a very small strip of ﬁlter paper (2 × 10 mm) was used as a bridge between the two rectangles of filter paper. Then an adult female spider mite was placed on the center of the bridge and mite movement was visually tracked. The filter paper over which the mite exited from the bridge was recorded, and the mite was removed. Subsequently, a new (naive) spider mite was placed on the bridge. Two bridges constructed side-by-side permitted testing one concentration of a solution with 30 mites in less than 30 min. For these 30 mites, the sums of the exits over treatment and over control rectangles, was expressed as a ratio - the exit ratio. Methods for calculation of response index and more details on the bioassay are provided in prior publications (Snyder et al., 2005; Snyder et al., 2011).

The bridge bioassay was used to evaluate oleoresins from the two LA2329 chemotypes as well as to evaluate compounds purified by chromatography. In an experiment one concentration of oleoresin or compound was tested with 30 individual mites, so there were 30 replications for each concentration tested. The experiment to evaluate oleoresins was conducted three times, and the experiment that evaluated purified compounds was conducted twice.

In bridge bioassays quantitation of compounds was based on GC-FID and only included abundance of the major components present in the oleoresin or separated fraction. Concentrations of oleoresins evaluated in the bridge bioassay ranged from 0.018 to 9 μg/cm²; concentrations of fractions obtained from silica gel chromatography were tested in the range of 0.001 to 0.2 µg/cm².

### Statistical Analysis

Statistical analyses were performed using the SAS Software package 9.4 (SAS Institute Inc., Cary, NC, USA). Quantities of trichome secretion components were analyzed by analysis of variance (ANOVA), and the means were compared using Duncan’s multiple range test. Exit ratios for each concentration of each fraction evaluated in the bridge bioassay were tested for homogeneity between the two bridges in the arena by χ^2^. In all cases, exit ratios were homogeneous between two bridges. EC_50_ and EC_90_ values were then estimated by use of PROC PROBIT, (SAS Institute 9.4 version), as described by Snyder et al. (2011). EC is an abbreviation for Effective Concentration, and an EC_50_ value is the predicted concentration at which 50% of the tested individuals respond to a stimulus; an EC_90_ value is the predicted concentration at which 90% of tested individuals respond.

## Results

### Composition and Abundance of Trichome Secretions on Two Chemotypes of LA2329 and on PI127826 and LA2167

**Figure 1:**
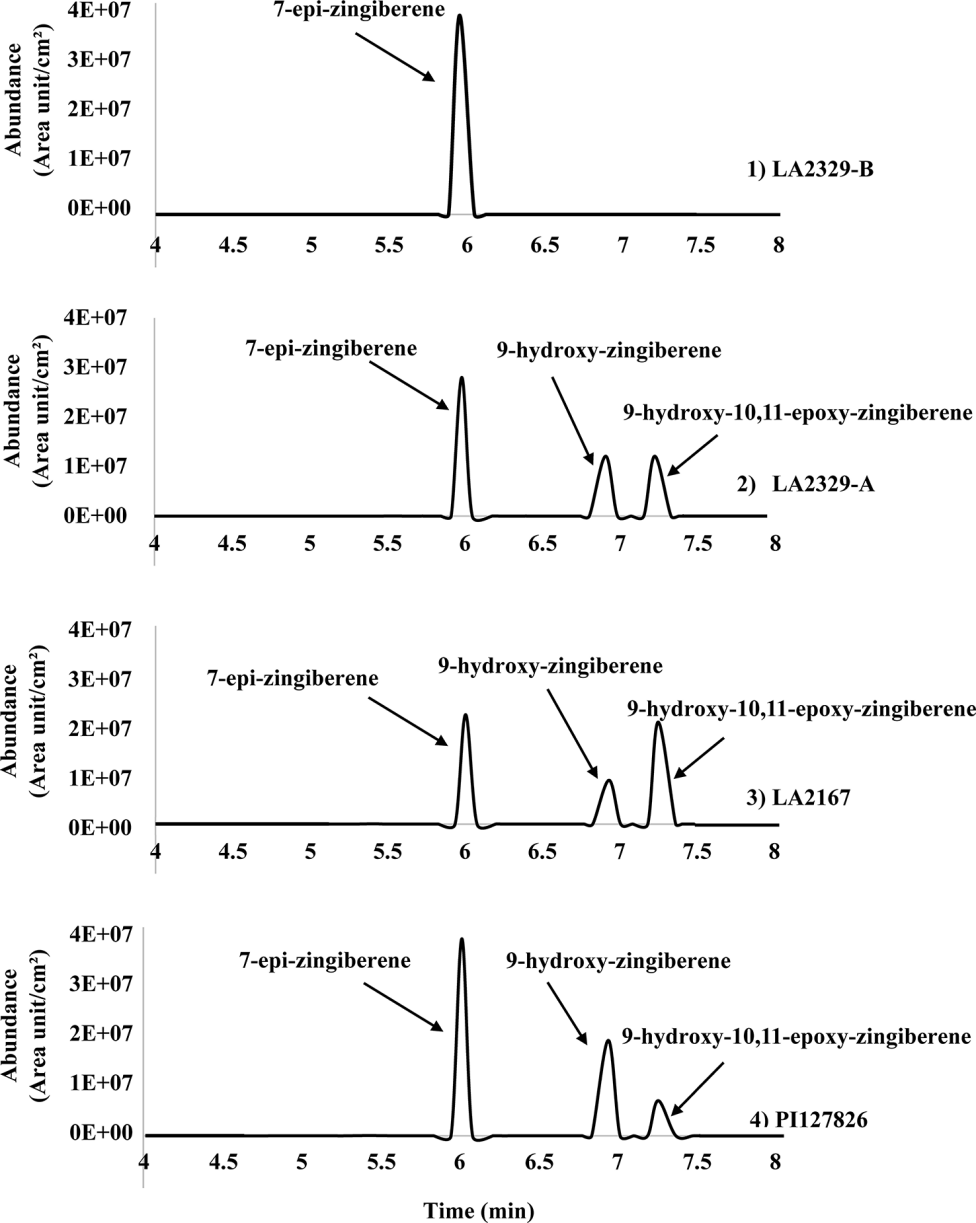
Chromatogram (GC-FID) of *n*-hexane leaflet wash from: (1) the LA2329-B chemotype showing a single major component at retention time of 6.00 min; (2) the LA2329-A chemotype showing three major components at retention times of 6.00, 6.91, and 7.23 min; (3) LA2167 showing three major components at retention times of 6.00, 6.91, and 7.23 min; (4) PI127826 showing three major components at retention times of 6.00, 6.91, and 7.23 min.

Three major compounds, **[1]**, **[2]**, and **[3]**, were present in *n*-hexane leaf washes LA2329, chemotype A (LA2329-A) (**Figure 1**). In contrast there was only one major component in washes of LA2329, chemotype B (LA2329-B) (**Figure 1**). The presence of this qualitative difference in trichome secretion composition between the two chemotypes supports the idea that the synthesis of secondary metabolites is very different in the two chemotypes. Furthermore, the GC-FID tracings for trichome secretions from LA2167 and PI127826 demonstrated the presence of three compounds each having a retention time that was identical to one of the three components present in LA2329-A. This indicates that the secretion composition of PI127826 and LA2167 was more similar to that on LA2329-A than to that on LA2329-B. In terms of the quality of trichome secretions, the quality of the secretion from LA2329-A was nearly the same as the trichome secretion qualities on PI127826 and LA2167, and was very different from the quality of the secretion on LA2329-B.

**Figure 2:**
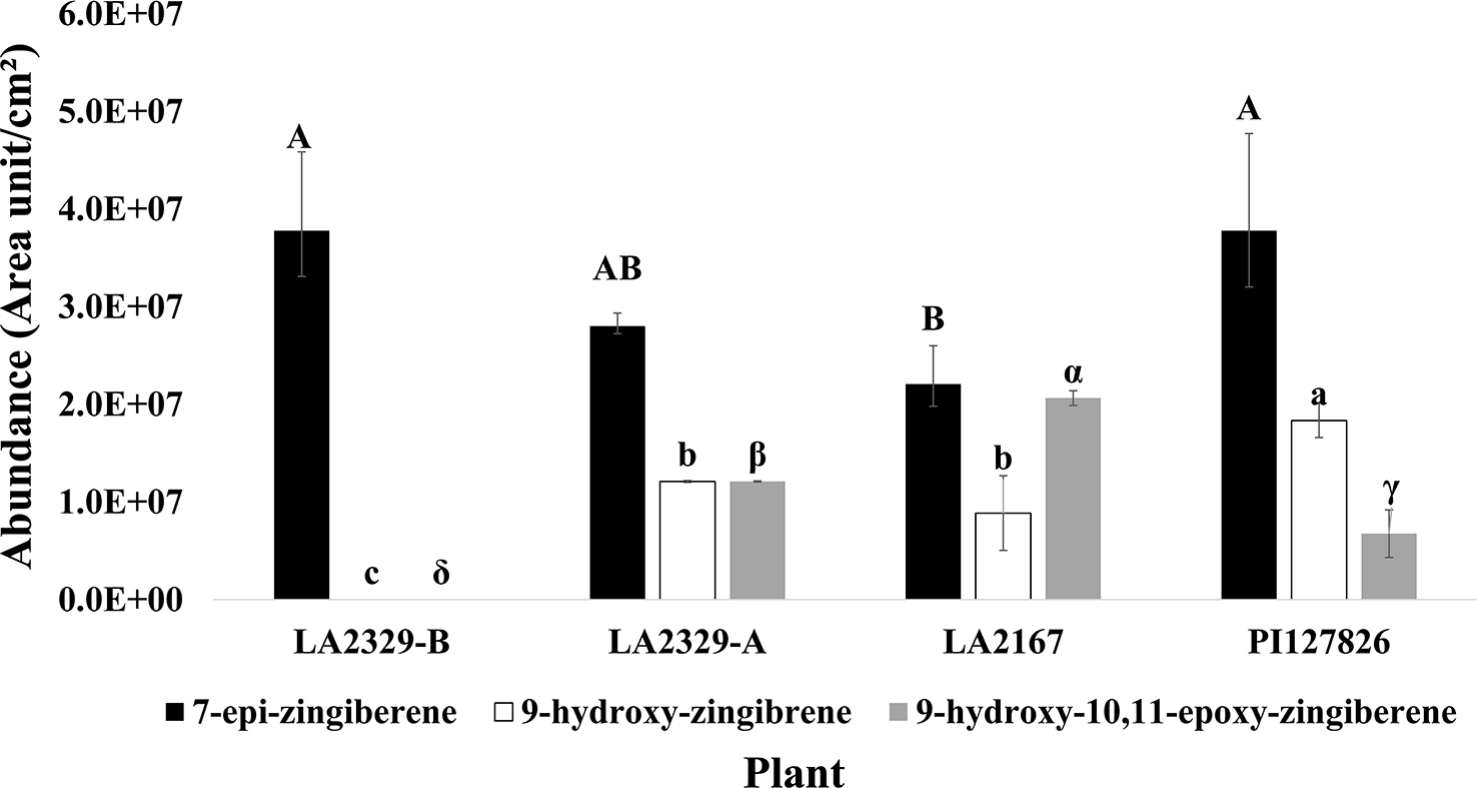
Abundance of 7-epi-zingiberene, 9-hydroxy-zingiberene, and 9-hydroxy,10-11-epoxy-zingiberene in four plants, two chemotypes of LA2329 (LA2329-A and LA2329-B), LA2167 and PI127826. Abundance of a compound labeled by the same letter(s) are not significantly different (P < 0.05).

When data for component abundances were analyzed by ANOVA, abundance of **[1]** (F = 6.15, P = 0.029), **[2]** (F = 61.87, P < 0.0001), and **[3]** (F = 42.25, P = 0.0002) varied significantly among plants (**Figure 2**).The abundance of **[1]** was highest in LA2329-A and PI127826, and lowest in LA2167. Concentration of [**1]** in LA2329-B was between these high and low concentrations. The abundance of **[2]** was highest in PI127826 and was not detected in LA2329-B. Concentrations of **[2]** in LA2329-A and LA2167 were significantly less than those observed on PI127826 and greater than that observed on LA2329-B. Abundance of **[3]** differed significantly among all four plants with concentration highest on LA2167 and not detected in LA2329-B. Based on the presence of significant differences of concentrations of **[1]**, **[2]**, and **[3]** among these plants **s**upports the idea that metabolic differences among the four plants could lead to quantitative differences in their trichome secretions.

### Repellent Activities of Oleoresins From LA2329 A and B Chemotypes

When oleoresins obtained from the two LA2329 chemotypes, A and B, were evaluated with the bridge bioassay, EC_50_ and EC_90_ values for the oleoresin from the B chemotype were considerably greater than that for the A chemotype (**Table 1**). In this assay, lower EC values indicate greater repellency so the EC value for the oleoresin having 3 major components from chemotype A (EC_50_ = 0.00709 µg/cm²) indicated an approximately six-fold greater repellent activity than that for oleoresin from chemotype B containing a single component (EC_50_ = 0.04791 µg/cm²).

**Table 1:** EC_50_ and EC_90_ values (µg/cm² of filter paper) and their 95% fiducial limits, standard deviations, and *P* values obtained from evaluation in the bridge bioassay of oleoresins collected from two chemotypes of *S habrochaites* LA2329.

Chemotype	EC_50_	95% Fiducial Limits		EC_90_	95% Fiducial Limits		Standard Deviation	P-Value
	(µg/cm²)	Lower	Upper	(µg/cm²)	Lower	Upper		
**A (Three components)**	0.00709	0.00513	0.00994	0.02561	0.02006	0.03567	0.0013	<.0001
**B (One component)**	0.04791	0.04975	0.05988	0.09716	0.08616	0.13398	0.0103	<.0001

The A chemotype contained three major components in its trichome secretions. The B chemotype had a single major component, **[1]**.

### Identification of 7-Epi-Zingiberene, 9-Hydroxy-Zingiberene, and 9-Hydroxy, 10,11-Epoxy-Zingiberene in LA2329 Chemotype A

**Figure 3:**
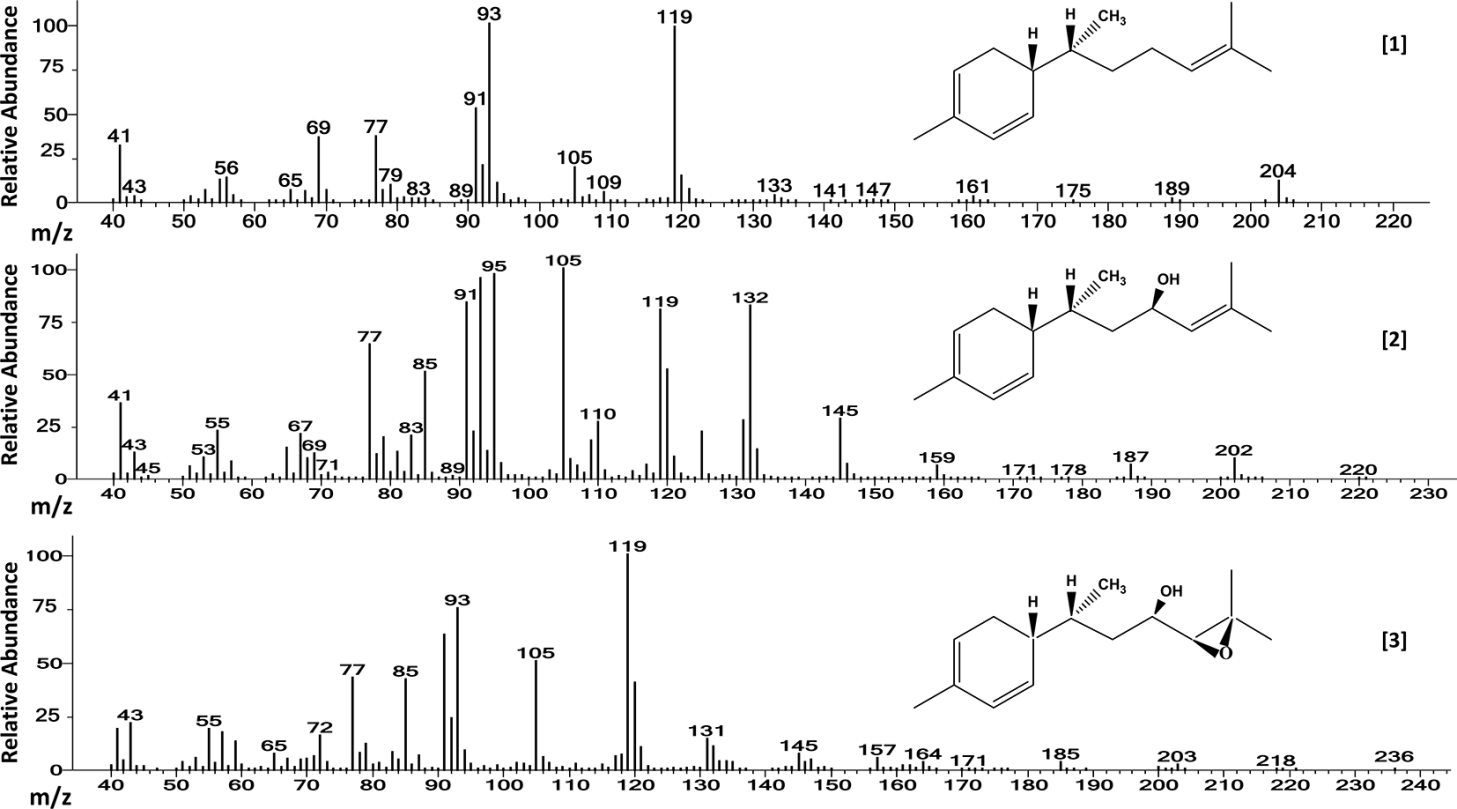
Electron ionization mass spectra of **[1]** 7-epi-zingiberene (C15H24), **[2]** 9-hydroxy-zingiberene (C15H24OH), and of **[3]** 9-hydroxy-10-11-epoxy-zingiberene (C15H24O2), present in trichome secretions of *S. habrochaites* accessions LA2329-A, LA2167, and PI127826. The names of three major components were obtained by comparing our results from GC-MS with those results published by Anonymous (2017) which was based on the analysis of trichome secretion components present on LA2167.

Mass spectra of the three major compounds present in extracts of the A chemotype of LA2329 wild tomato accession showed a molecular ion (M+) at m/z 204 for **[1]**. For **[2]** the molecular ion (M+) was at m/z 220. And for **[3]**, the molecular ion (M+) was located at m/z 236. These outcomes were consistent with a sesquiterpene hydrocarbon (C15H24), and sesquiterpenoids having empirical formulas of C15H24O and C15H24O2, respectively (**Table 2** and **Figure 3**). Identical spectra (99% match) and retention times were obtained for each of the three major compounds present in leaf washes obtained from PI127826 and LA2167. Because of a complete match of retention times and spectra among the three accessions for the three compounds, we concluded that the identities of the three peaks present in LA2329-A were 7-epi-zingiberene **[1]**, 9-hydroxy-zingiberene (**[2]**), and 9-hydroxy-10,11-epoxy-zingiberene (**[3]**).

**Table 2:** GC-FID and GC-MS retention times, molecular ion (M+) and characteristic mass fragments of the three allelochemical components extracted from wild tomato accessions *Solanum habrochaites* (LA2329, PI127826, and LA2167).

Compound	Retention time (RT)		Molecular ions (M+), Fragments, and (Relative Abundance)
	GC-FID	GC-MS	
**[1] 7-epi-zingiberene**	6.00	8.44	204 (M+), 93 (99), 91 (52), 77 (37), 69 (36), 41(32), 92 (21), 105 (19), 120 (15), 56 (13), 55 (13)
**[2] 9-hydroxy-zingiberene**	6.91	9.45	220 (M+), 105 (100), 95 (97), 93 (95), 91 (84), 132 (82), 119 (80), 77 (64), 120 (52), 85 (51) 41 (36)
**[3] 9-hydroxy-10,11-epoxy-zingiberene**	7.22	9.88	236 (M+), 119 (100), 93 (75), 91 (63), 105 (51), 77 (43), 85 (42), 120 (40), 92 (24), 43 (21), 55 (19)

Fragments are listed by m/z followed by relative abundance in parentheses.

### Silica Gel Chromatography of LA2329 Chemotype A Oleoresin

The three compounds were well separated by silica gel chromatography**. [1]** was present in fractions 3 to 6 at purities, judged by GC-FID, ranging from 90% to 98% and concentrations ranging from 1 to 10 µg/mL. **[2]** was present in fractions 32 to 37 at purities ranging from 78% to 86% and concentrations ranging from 0.4 to 4.5 µg/mL and **[3]** was present in fractions 50 to 53 at purities ranging from 83% to 87% and concentrations ranging from 0.3 to 0.9 µg/mL. Fractions for bioassay were chosen based on the purity of the fraction. This minimized the influence of the presence of non-target chemicals on the bioassay results. Thus, fractions 4 and 5 for **[1]**, fractions 33 and 34 for **[2]**, and fractions 51 and 52 for **[3]** were chosen for evaluation of repellency in the bridge bioassay.

Repellent activity of the isolated **[1]** as judged by EC_50_ and EC_90_ values obtained with the bridge bioassay was highest of the three compounds evaluated, indicating that **[1]** was least repellent of the three compounds tested (**Table 3**). EC_50_ and EC_90_ values for **[2]** and **[3]** were 5 to 10-fold less than that for **[1]**, indicating that these two oxygen-containing sesquiterpenoids were considerably more repellent than the hydrocarbon **[1]**. In other words, compared to **[1]**, much smaller doses of **[2]** or **[3]** were required to repel spider mites, compared to **[1]**. The EC_50_ and EC_90_ estimates from probit analysis of data had narrow fiducial limits and non-significant χ^2^ values for lack of fit. Thus, the bioassays provided highly precise estimates of effective concentrations for the repellency of the tested compounds. Fiducial limits for the oxygen-containing sesquiterpenoids **[2]** and **[3]** did not overlap those for the hydrocarbon **[1]**, supporting the conclusion that **[1]** was less repellent in the bridge bioassay than the two oxygen-containing sesquiterpenoids, **[2]** and **[3]**. However, for these two sesquiterpenoids, fiducial limits did overlap, indicating that EC values and repellent activities of these two compounds, **[2]** and **[3]** were not differentiated by the bridge bioassay. In other words, the EC values and consequently the repellency of **[2]** and **[3]** were not separated statistically in this experiment.

**Table 3:** EC_50_ and EC_90_ values (µg/cm² of filter paper) and their 95% fiducial limits, standard deviations, and *P* values of three allelochemicals ([1], [2], and [3]) isolated by silica gel chromatography and tested in the bridge bioassay.

Allelochemical	EC_50_	95% Fiducial Limits		EC_90_	95% Fiducial Limits		Standard deviation	P-Value
	(µg/cm²)	Lower	Upper	(µg/cm²)	Lower	Upper		
**[1] 7-epi-zingiberene**	0.01655	0.01392	0.0198	0.0481	0.03785	0.06612	0.00030	<.0001
**[2] 9-hydroxy-zingiberene**	0.00198	0.00165	0.00235	0.0073	0.0057	0.00103	0.00032	<.0001
**[3] 9-hydroxy,10,11-epoxy-zingiberene**	0.00182	0.00167	0.00216	0.00448	0.00363	0.00602	0.00018	<.0001

## Discussion

Wild tomato accessions such as those of *S.h.* are great potential sources of insect and disease resistance for tomato breeding (Vidavski, 2007; Bleeker et al., 2012), partly because many of these accessions contain high levels of allelochemicals in their trichomes. One such allelochemical is 7-epi-zingiberene **[1]** which was present on both chemotypes of LA2329. The oleoresin from LA2329 chemotype B, which contained a single major component, **[1]** was repellent to spider mites in the bridge bioassay. However, the oleoresin from LA2329 chemotype A, having three major components, had considerably greater repellency than the oleoresin containing one major component. This observation supports the hypothesis that compounds other than **[1]** that were present in LA2329 chemotype A, namely compounds **[2]** and **[3]** are likely much more repellent to spider mites than **[1]**.

We verified by GC-MS the identities of the major components present in LA2329 chemotype A. **[1]** was present as were **[2]** and **[3]**. Mass spectra and retention times completely matched those obtained for authentic compounds isolated from LA2167 (Anonymous, 2017). **[2]** and **[3]** were also present in trichome secretions from the widely studied *S.h*. accession PI127826.

**[1]**, **[2]** and **[3]** were successfully separated by silica gel chromatography. Quantities and purities of allelochemicals isolated by chromatography were sufficient to permit evaluation of spider mite repellency of each compound in bridge bioassays. The repellent activities of the two oxygenated components, **[2]** and **[3]**, based on their EC_50_ and EC_90_ values were 6 to 10 – fold more repellent than **[1]**. This result confirms our hypothesis that these two oxygenated sesquiterpenoids **[2]** and **[3]** are more repellent to spider mites than the sesquiterpene hydrocarbon, **[1]**.

The results presented herein are in agreement with those of Guo et al. (1993). These authors concluded that the greater repellencies of trichome secretions on certain accessions of *S.h*. are likely due to the presence of compounds that are more polar than sesquiterpene hydrocarbons; **[2]** and **[3]** are each more polar than their parent sesquiterpene hydrocarbon, **[1]** and were more repellent in bridge bioassays than **[1]**. While we did not evaluate insect antibiotic properties of these molecules, it is possible that these oxygenated compounds may also have contributed to the strong antibiosis to beet army worm (*Spodoptera exigua)* of trichome secretions from *S.h*. LA2329 observed by Eigenbrode et al. (1994). Furthermore, because we have reported that **[2]** and **[3]** are present in trichome secretions of *S.h*. PI127826, conclusions that impute causes of resistance in this accession solely to the presence of the sesquiterpene hydrocarbon **[1]** need reconsideration (Maluf et al., 2001; de Azevedo et al., 2003).

In addition to spider mite repellency, oxygenated sesquiterpenoids present in trichome secretions may play a role in mediating interactions of *S.h.* with other arthropods. For example, sesquiterpenoid acids from the *S.h*. accession LA1777 reduce larval feeding and survival of *Heliocoverpa zea* and *Spodoptea exigua* (Frelichowski Jr and Juvik, 2001) and surprisingly, stimulate oviposition of *H. zea* (Coates et al., 1988). The occurrence of oxygenated sesquiterpenoid derivatives needs to be surveyed in this diverse wild tomato species, as does their biochemical origins in the plant and their impact of behavior of significant arthropod pests such as the virus vector *Bemesia* spp. all need additional evaluation. Antixenosis which seems to be present in many accessions of the *S.h.* species should be emphasized (Guo et al., 1993; Snyder et al., 1993; Eigenbrode et al., 1996; Snyder et al., 1998).

**Figure 4:**
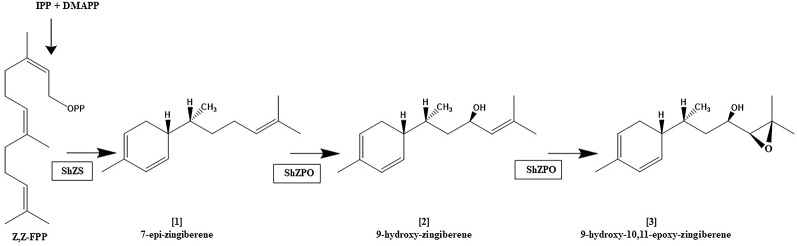
Proposed biosynthetic pathway for the zingiberene derivatives present in trichome secretion of S. *habrochaites* accession LA2167. zFPS: Z,Z-FPP synthase, ShZS: zingiberene synthase (also referred to herein as ShZIS), ShZPO, zingiberene polyoxidase. This figure is based on information in Anonymous (2017).

It appears that not only are terpene profiles variable in *S.h,* the presence of oxygenated sesquiterpenoids in trichome secretions may also be variable. The trichome secretions on the *S.h.* accession LA1777 are predominated by the presence of alpha-santalenoic and endo-beta-bergamotenoic acids (Coates et al., 1988) and those on LA1363 are predominated by the presence of 2,3-dihydrofarnesoic acid (Guo et al., 1993). The biosynthetic origin of these oxygenated forms has not be delineated, but is likely the consequence of action by a cytochrome P450 on a parent sesquiterpene hydrocarbon, similar to the action the of the cytochrome P450 monooxygenase, CYP71AV1 in *Artemisia annua* that performs a three-step oxidation of the sesquiterpene hydrocarbon amorpha-4,11-diene to artemisinic acid (Ro et al., 2006). The conversion of **[1]** into **[2]** and **[2]** into **[3]** in trichomes, as depicted in **Figure 4**, is reportedly also the result of action of a cytochrome P450 named zingiberene polyoxidase (Anonymous, 2017). The apparent variation in the ratio of abundance of **[1]**, **[2]** and **[3]** among LA2329-A, LA2167, and PI127826 (**Figure 2**) supports the idea that there may be considerable differences in the action of zingiberene polyoxidase among these three accessions.

Our research was aided considerably by the presence of two chemotypes in our population of *S.h*. LA2329. That we uncovered variation in trichome secretion composition within LA2329 is not surprising because it has been noted that there is more genetic variability within a single accession of self-incompatible wild tomato such LA2329 than there is in all of cultivated tomato (Bai and Lindhout, 2007). It is likely that a systematic search of the other wild tomato accessions would uncover chemical diversity in their trichome secretions. Also, it is likely that variation for presence of **[2]** and **[3]** in our population of LA2329 was directly connected to variation associated with the zingiberene polyoxidase locus.

Because of the apparent greater repellent activity of the oxygenated sesquiterpenoids, **[2]** and **[3],** plant breeders should consider introgression of these compounds into cultivated tomato, with a view toward additional evaluation of the role of these compounds in arthropod resistance of tomato. Hopefully, tomato plants that produce these more repellent compounds would demonstrate greater resistance to spider mites, and perhaps, to other arthropods.

Introgression of zingiberene oxidase may be a relatively straightforward breeding task. Likely a single enzyme, a cytochrome P450, is responsible for producing these two compounds from 7-epi-zingiberene (Anonymous, 2017). Thus, introgression may be particularly direct when using a recurrent parent such as one of our advanced breeding lines that produce high concentrations of **[1]** in their trichomes. Introgression of the ability to produce the alcohol **[2]** and epoxide **[3]** may also lead to greater production of total sesquiterpenoids on tomato, similar to the difference of sesquiterpenoid production between the LA2329-A and LA2329-B chemotypes (**Figure 2**).

The bridge or springboard bioassay has been demonstrated as sufficiently sensitive to detect differences in repellency of molecules having subtle structural differences, such as the presence or absence of a double bond (Snyder et al., 2011). The results reported here support the conclusion that the bridge bioassay can be used to demonstrate differences of repellency between a sesquiterpene hydrocarbon, **[1]**, and two oxygenated derivatives of zingiberene, **[2]** and **[3]**. However, this bioassay did not demonstrate any difference in repellent activity between the two oxygenated derivatives of **[1]**.

Volatile substances present in plant glandular trichomes can prevent and/or reduce interactions with arthropod vectors of viral diseases (Aharoni et al., 2005), thereby reducing disease incidence. For example, the presence of exogenously applied **[1]** seems to repel the whitefly (*Bemisia tabaci*) from tomato plants. Because *B. tabaci* is a begomovirus vector (Rosen et al., 2015), Bleeker et al. (2012) suggested that introduction of the biosynthetic pathway for production of **[1]** would provide protection against virus transmission. Introduction of the ability of the plant to produce **[2]** and/or **[3]** could be even more beneficial if these oxygenated forms of zingiberene are more effective than **[1]** in repelling whiteflies, similar to our findings with TSSM.

Some wild tomato species are known as having very high levels of arthropod resistance (Rick, 1984; Simmons and Gurr, 2005; Barrantes et al., 2016). While the existence of these genetic resources is valuable, complete delineation of a resistant phenotype for a highly resistant individual can be difficult. For example, evaluation by bioassay of causes of resistance in a plant having high levels of antixenosis could mask mechanisms of antibiosis. Likewise, presence of multiple active compounds such as in the A chemotype of LA2329, can considerably complicate delineation of causes of resistance. Although there are many wild sources of resistance to arthropods, the lack of released varieties having high levels of resistance is a good indication of difficulty of using these genetic resources.

Results of the research have implications not only for tomato breeders but also for chemical ecologists, and plant evolutionists as well. This research was prompted by the presence of two chemotypes within our locally maintained population of LA2329 and an observation that one chemotype appeared more resistant to whitefly than the other. According to the Tomato Genetics Resource Center (tgrc.ucdavis.edu) the *S.h*. accession LA2329 is allogamous-self-incompatible. However, our population of LA2329 was established from just a few plants, and because of this, as we have produced subsequent seed generations, we have also likely inbred some of the plants which could have allowed expression of recessive genes. That we uncovered variation for composition of trichome secretions within an accession of *S.h*. is not surprising, given the genetic variability available in wild tomato accessions (Bai and Lindhout, 2007). We believe that this is the first report of qualitative variation of trichome secretion composition within an accession of *S.h.* Furthermore, it is likely that the chemotypic variability observed in our population of LA2329 is associated with the variability in the expression or activity of the P450 oxidase responsible for production of oxygenated sesquiterpenoids **[2]** and **[3]** as described by Anonymous (2017). Furthermore, given the extensive genetic variability that exists within wild tomato accessions, it is likely that components of trichome secretions may be variable within other accessions of *S.h*. The potential for variability of sesquiterpenoids in trichome sections of *S.h*. should attract research interest by chemical ecologists, plant evolutionists as well at those interested practical aspects of plant-arthropod interactions, such as entomologists and plant breeders.

In conclusion, the preparative separation and purification of **[1]**, **[2]** and **[3]** were successfully accomplished. After identification and purification, the three major allelochemical components were tested for TSSM repellency in the bridge bioassay. All allelochemical components repelled spider mites. Yet, the activities of **[2]** and **[3]** were both much higher than that for **[1]**. Future research should center on introgression of these sesquiterpenoids into cultivated tomato and evaluation of their antibiotic and antixenotic activities on arthropods other than TSSM. Future studies should also utilize these three allelochemical components to minimize pesticide use and guarantee long-term pest management.

## Data Availability Statement

The raw data supporting the conclusions of this article will be made available by the authors, without undue reservation, to any qualified researcher.

## Author Contributions

MD conducted the experiments, performed the laboratory experiment by separating and purifying the allelochemical compounds, performed the bioassay tests and wrote the manuscript. JS analyzed the results, managed the data, contributed the experimental design and wrote the manuscript. Before submission, both authors read and approved the final manuscript.

## Funding

Partial funding for this work was provided through the Kentucky Agriculture Experiment Station Hatch Project KY011044.

## Conflict of Interest

The authors declare that the research was conducted in the absence of any commercial or financial relationships that could be construed as a potential conflict of interest.

## Supplementary Material

Supplementary Figure S1 Bridge bioassay arenas used for repellency bioassays. Each consisted of two small rectangles (0.75 cm² × 1 cm) of filter paper that were bridged by a very small strip of filter paper (1 cm² × 2 mm). Filter paper rectangles labeled T were treated with a known concentration of test compound(s), while those labeled C were loaded with solvent (n-hexane) only.

## Abbreviations

*S.h*, *Solanum habrochaites*; **[1]**, 7-epi-zingiberene; **[2]**, 9-hydroxy-zingiberene; **[3]**, 9-hydroxy-10,11-epoxy-zingiberene; MTBE, methyl-tert-butyl ether; TSSM, two-spotted spider mite; GC-FID, Gas Chromatography - Flame Ionization Detector; GC-MS, Gas Chromatography Mass Spectroscopy.
